# Associations between psychological symptoms in adolescence and debts or experienced financial scarcity in emerging adulthood

**DOI:** 10.1192/bjo.2025.10844

**Published:** 2025-09-19

**Authors:** Susan J. Ravensbergen, Nina H. Grootendorst-van Mil, Richard Wesseloo, Witte J. G. Hoogendijk, Diandra C. Bouter

**Affiliations:** Department of Psychiatry, Erasmus MC University Medical Center Rotterdam, Rotterdam, The Netherlands; Epidemiological and Social Psychiatric Research Institute, Department of Psychiatry, Erasmus MC University Medical Center, Rotterdam, The Netherlands; Bipolar Disorder Expert Team, GGZ Delfland, Delft, The Netherlands

**Keywords:** High risk, financial stress, development, psychiatric problems

## Abstract

**Background:**

Patients with psychiatric disorders are more likely to experience financial difficulties. Yet, there is limited knowledge about the longitudinal relationship between psychopathology in adolescence and debts or experienced financial scarcity in emerging adulthood.

**Aims:**

We aimed to examine whether distinct types of psychological symptoms in mid-adolescence precede the occurrence of unsecured debts and experienced financial scarcity in emerging adulthood.

**Method:**

Data from a Dutch adolescent cohort (*N* = 659, 53.9% female) oversampled on the risk of psychopathology was used. Psychological symptoms were self-reported at baseline (mean age 14.80 years, s.d. = 0.79), using the Youth Self-Report and the Prodromal Questionnaire-16. At follow-up (mean age 17.95 years, s.d. = 0.80), adolescents reported presence of unsecured debts and completed the Psychological Inventory of Financial Scarcity. Logistic and linear regression analyses were applied, adjusting for the baseline characteristics of sex, age, ethnic background, parental psychopathology and household income as an indicator of family socioeconomic status.

**Results:**

More attention-deficit/hyperactivity problems (adjusted odds ratio 1.15, 95 CI% 1.04–1.27), oppositional defiant problems (adjusted odds ratio 1.17, 95 CI% 1.00–1.35) and psychotic experiences (adjusted odds ratio 1.10, 95 CI% 1.01–1.21) at age 15 years were associated with unsecured debts at age 18 years. Depressive, anxiety, somatic and conduct problems were not associated with later unsecured debts. Depressive, anxiety, attention-deficit/hyperactivity, oppositional defiant, conduct problems and psychotic experiences at age 15 years were associated with increased experienced financial scarcity at age 18 years.

**Conclusions:**

Psychological symptoms during mid-adolescence precede both objective and subjective financial difficulties when entering adulthood. Integrating financial education into prevention efforts could potentially provide significant long-term benefits, particularly for those with mental health problems.

Reaching the age of 18 marks a significant milestone for the formal transition to adulthood. In Europe, adolescents at this age gain full legal responsibility for their financial actions.^1^ They gain the ability to open credit card accounts, borrow money from financial institutions and many students start a student debt. This increase in autonomy, combined with not fully matured decision-making skills,^2^ may put adolescents at risk for making poor financial choices that could have long-term consequences. For example, research suggested that student debts may contribute to future overspending^3^ and can affect the future disposable income for up to 35 years.^4^ Therefore, it is essential to identify adolescents who may be at risk of developing financial difficulties, to offer them targeted financial education.

## Debt and mental health

A potential risk factor for such financial difficulties is mental health issues. A systematic review and meta-analysis from Richardson et al^5^ revealed a clear connection between financial problems and mental health issues in adults. Unsecured debts, which are not backed by collateral, were associated with higher prevalences of mental disorders, such as depression, suicidality and psychotic disorders, with odds ratios ranging from 2.8 to 8.6. Yet, the authors note a lack of longitudinal evidence, which leaves the question of causality unresolved.^5^ A few longitudinal studies in adulthood have found evidence for increasing debts and subsequent worsening of mental health.^6^ A longitudinal study involving young adults aged 18–29 years revealed that an increase in debt during one period was associated with poorer mental health in the next period.^7^ However, most of these studies did not control for prior history of mental health problems or other socioeconomic variables, nor did they test whether psychopathology symptoms increased the risk of financial difficulties.^6^ Additionally, the majority of these studies assume that financial problems cause mental health issues, in line with the social causation hypothesis,^8^ whereas psychiatric symptoms may have been present before the onset of financial problems. For example, when considering behavioural problems in childhood, specifically criminal behaviour and attention-deficit/hyperactivity, it has been demonstrated that this is associated with worse financial outcomes in adulthood.^9–12^

Examining the relation from this alternative perspective, it is known that early psychopathology is a well-known risk factor for numerous adverse life outcomes. Adolescents experiencing mental health problems, even in the absence of a full-blown diagnosis, are particularly vulnerable to various adverse outcomes, such as poorer academic or occupational performance, and low income.^13–15^ This is further underlined by the health selection or social drift hypothesis, both explaining how poor health will often lead to poor socioeconomic positions.^8,16^ Together, this underscores the necessity of examining the relation in this direction, suggesting that psychopathology may precede financial difficulties. The stage of adolescence is particularly interesting to further examine this, given that it is a crucial period for physical, emotional, psychological and cognitive growth,^17^ and includes the starting point for legal financial responsibility. Moreover, most adult psychiatric disorders can be traced back to adolescence, where half of the disorders first emerge.^18^

## Subjective financial difficulties

In addition to objective financial problems like debts, it has been proposed that individuals’ subjective evaluation of their financial situation could mediate the relation between debts and health, or may hold greater importance than the objective financial situation when linked to health.^5,19,20^ Again, there is limited understanding regarding the longitudinal relation between psychopathology and subjective financial difficulties, especially beginning in adolescence. Among the few existing studies, a longitudinal study in a university sample found that financial difficulties seemed to lead to mental health problems, with the suggestion of a bi-directional relation.^21^ Following the same reasoning as with objective financial problems, adolescent psychopathology may also serve as a risk factor for subjectively perceived financial difficulties in emerging adulthood. Additionally, internalising disorders, such as depression or anxiety, are particularly characterised by worrying and cognitive distortions.^22,23^ As adolescents transition into adulthood, this may also manifest in negative thoughts about their financial situation, which eventually might exacerbate existing mental health problems.

The present study aimed to examine whether particular self-reported psychological symptoms during mid-adolescence (age 15 years) precede the occurrence of unsecured debts or experienced financial scarcity in emerging adulthood (age 18 years), adjusting for important confounders such as family socioeconomic status. We assessed depressive, anxiety, somatic, attention deficit/hyperactivity, oppositional defiant and conduct problems, and psychotic experiences. Based on previous studies showing a relation between financial difficulties and various mental disorders, we expected all psychological symptoms to be related to future debts and experienced financial scarcity. These results may inform strategies for targeting specific adolescent groups for enhanced financial education, either integrated with mental healthcare or in educational settings.

## Method

### Procedure and participants

This study was embedded in the iBerry Study, a prospective cohort study in the Greater Rotterdam area (The Netherlands), aimed at detecting determinants for the development of psychopathology.^24^ As part of a general routine healthcare check, adolescents filled in the Strengths and Difficulties Questionnaire – Youth (SDQ-Y) during their first year of high school. From those who did not object to study participation, a selection was created based on their SDQ-Y score. Adolescents who scored within the highest 15% and a random selection of the remaining adolescents were approached for participation in the iBerry Study (mean age at screening 13.1 years). In total, 1022 adolescents (mean age 15.0 years, 51.1% female) participated in the baseline measurement conducted between 2015 and 2019, with an intentional oversampling of high SDQ-Y scores (2.5:1 ratio), resulting in a cohort with an increased risk for developing psychopathology. Further details on the screening procedure and response are provided by Grootendorst-van Mil and Bouter et al.^24^ The first follow-up measurement (2019–2022) was conducted approximately 3 years after baseline (*n* = 807 adolescents, mean age 18.1 years, 53.5% female). Details regarding drop-out and the development of psychopathology are described by Bouter et al.^25^ At both baseline and follow-up, adolescents and an accompanying parent completed various (psychiatric) interviews, questionnaires, cognitive tasks and physical measurements. All participants (and legal guardians, if applicable) provided written informed consent.

The authors assert that all procedures contributing to this work comply with the ethical standards of the relevant national and institutional committees on human experimentation and with the Helsinki Declaration of 1975, as revised in 2013. All procedures involving human participants were approved by the Medical Ethics Review Committee of the Erasmus MC, University Medical Center Rotterdam (approval numbers MEC 2015-007 and MEC 2018-1472).

### Measurements

#### Self-reported psychological symptoms

The Youth Self-Report (YSR 11-18)^26,27^ was administered to the adolescent at the baseline measurement. This 112-item self-report questionnaire asks about emotional and behavioural problems over the past 6 months. Example items are ‘I have trouble concentrating or paying attention’ and ‘I feel worthless or inferior’. Answers can be given on a three-point scale ranging from 0 (‘not true) to 2 (‘very often or often true’). Following one of the scoring methods, scores were summed into the following DSM-5-oriented subscales: Depressive Problems, Anxiety Problems, Somatic Problems, Attention-Deficit/Hyperactivity Problems, Oppositional Defiant Problems and Conduct Problems. Higher scores represented more problems. Although the subscales refer to the DSM-5, these self-report scales assess subjective symptoms and distress, rather than formal clinical diagnoses. The YSR has been validated, and reliability has been demonstrated in this age group.^27^ In our sample, Cronbach’s alphas generally indicated acceptable internal consistency (ranging from 0.70 to 0.77). For the Oppositional Defiant Problems and Conduct Problems subscales, there was questionable internal consistency, with Cronbach’s alphas of 0.62 and 0.67, respectively.

To assess psychotic experiences at baseline, we used the Prodromal Questionnaire-16 (PQ-16).^28^ Within 16 self-reported items, the adolescent was asked about both hallucinatory and delusional experiences; for example, ‘I have seen things that other people apparently can’t see’ and ‘I sometimes see special meanings in advertisements, shop windows or in the way things are arranged around me’. Each experience was answered on a dichotomous scale (0 = ‘no’, 1 = ‘yes’). Items were summed, with higher scores indicating more psychotic experiences. The PQ-16 is a validated screening tool to assess psychosis risk.^28^ The internal consistency in our sample was acceptable (Cronbach’s alpha of 0.77).

#### Unsecured debts and experienced financial scarcity

At follow-up, we created a questionnaire in collaboration with the Dutch National Institute for Family Finance Information (Nibud), to assess the current financial situation of the adolescents. Within this questionnaire, adolescents were asked whether they had unsecured debts (yes/no), and if they did, they were asked to specify the type and amount of debt. Furthermore, a shortened version of the Psychological Inventory of Financial Scarcity (PIFS)^29^ was incorporated in the questionnaire to measure current experienced financial scarcity. The adolescent was asked six items about their perception, worries and control regarding their financial situation. Items include ‘I am constantly wondering whether I have enough money’ and ‘I experience little control over my financial situation’. Answers were given on a three-point scale ranging from 0 (‘disagree’) to 2 (‘agree’), and a total score was computed. Higher scores corresponded with more experienced financial scarcity. The PIFS has been validated, and its reliability has been established in previous research.^29^ A Cronbach’s alpha of 0.79 demonstrated acceptable internal consistency in our sample.

#### Sociodemographic characteristics

Adolescents and an accompanying parent provided details on sociodemographic characteristics at baseline, which included age, sex and ethnic background. Ethnic background was dichotomised into Dutch and non-Dutch, determined by the country of birth of the adolescent and their parents. To include an indicator of socioeconomic status, the parent reported the net monthly household income, which was categorised into <€1600, €1600–€2399, €2400–€4399, and ≥€4400.

#### Estimated intelligence

As financial literacy has been linked to general intelligence,^30^ we added IQ score as a covariate in our model. The estimated IQ score of the adolescent was determined at baseline by their performance on the Analogies and Categories subtests of the Snijders-Oomen Nonverbal Intelligence Test (SON-R 6-40).^31^ Scores were doubled to correspond with the test’s original structure, and were then corrected for the Flynn effect.

#### Parental psychopathology

The accompanying parent completed the Brief Symptom Inventory (BSI)^32^ at baseline to provide details on parental psychopathology. Each item was answered on a three-point scale ranging from 0 (‘not at all or little’) to 2 (’often’). The total score of the 53 items was used, with higher scores representing more psychological symptoms. A Cronbach’s alpha of 0.94 indicated excellent internal consistency.

### Statistical analyses

#### Missing data

Adolescents were included in our sample if data was available for at least one psychological symptoms and at least one financial measure (*n* = 744). Missing items on a subscale were substituted with the participant mean subscale item score, up to 25% missing. Missing covariates included ethnic background (0.1%), IQ score (4.0%), parental psychopathology (12.0%) and household income (10.1%). The complete case sample was compared with adolescents who had missing scores on at least one covariate (see Supplementary Table 1 available at https://doi.org/10.1192/bjo.2025.10844). The samples differed on several covariates and the occurrence of debts (outcome). As the missingness of the covariates seemed to be related to whether the parent participated or not, the missing (completely) at random assumption for multiple imputations could potentially be violated. In the absence of adequate auxiliary variables, we used the complete case sample (*n* = 659), as regression coefficients of the complete-case analyses would likely be unbiased.^33^

#### Analyses

Hierarchical regression analyses were performed to assess the relation between self-reported psychological symptoms at baseline and financial difficulties at follow-up. Logistic regression analyses were applied regarding the occurrence of debts, and linear regression analyses were performed for experienced financial scarcity. The YSR DSM-oriented scores and the PQ-16 sum score were used as predictors. Covariates were entered hierarchically. In model 1, we adjusted for sex and age only, to examine the crude effect. In model 2, we added ethnic background, IQ score and parental psychopathology to adjust for demographic and parental factors. In model 3, the fully adjusted model, we entered household income, to test whether any associations between psychological symptoms and financial difficulties could be attributed to a low socioeconomic status of the family. For the logistic regression analyses predicting debts by somatic problems, the assumption of linearity of the logit was violated, which was solved by applying a square root transformation of the somatic problems score. Within all analyses where experienced financial scarcity was the outcome variable, the normality assumption was violated and a square root transformation to the experienced financial scarcity score was applied to address this. A *P*-value of <0.05 was considered significant. All analyses were performed in IBM SPSS statistics (version 28.0.1.0 on Windows 10). To correct for multiple testing, we used the false discovery rate correction proposed by Storey^34^ to calculate *q*-values from the given *P*-values. This method balances the risk of type one and type two error. Because of the difference in *P*-value distributions across the two outcome measures, *q*-values were calculated independently for each outcome measure. The *q*-value package in R (version 4.3.2 on Windows 10) was used,^35^ and a *q*-value of <0.05 was considered significant.

## Results

Table 1 presents the sample characteristics at baseline. The study sample consisted of 659 adolescents with a mean age of 14.80 years at baseline and a mean age of 17.95 years at follow-up. Slightly more females than males were included in the study (53.9%). Most adolescents were of Dutch descent (80.3%). At follow-up, 51 (7.8%) adolescents reported being in debt, with a median debt of €390 (25th percentile: €108, 75th percentile: €1350). Most of them had a student loan (41.2%), followed by borrowing money from others (19.6%) and falling behind with payments (9.8%).

**Table 1:** Characteristics and symptoms of the adolescents included in the sample

	Total sample (*n* = 659)
Age at baseline, years, mean (s.d.)	14.80 (0.79)
Age at follow-up, years, mean (s.d.)	17.95 (0.80)
Sex, female, *n* (%)	355 (53.9)
Ethnic background, Dutch, *n* (%)	529 (80.3)
IQ score, mean (s.d.)	99.55 (13.56)
Household net monthly income, *n* (%)	
<€1600	60 (9.1)
€1600–2399	97 (14.7)
€2400–4399	349 (53.0)
≥€4400	153 (23.2)
Parental psychopathology, score, median (IQR)	5.00 (10.0)
Depressive problems, score, median (IQR)	4.00 (5.00)
Anxiety problems, score, median (IQR)	3.00 (4.00)
Somatic problems, score, median (IQR)	2.00 (4.00)
Attention deficit/hyperactivity problems, score, median (IQR)	7.00 (4.00)
Oppositional defiant problems, score, median (IQR)	2.00 (3.00)
Conduct problems, score, median (IQR)	3.00 (3.00)
Psychotic experiences, median (IQR)	3.00 (4.00)
Debt present at follow-up, yes, *n* (%)	51 (7.8)
Experienced financial scarcity at follow-up, score, median (IQR)	2.00 (4.00)

IQR, interquartile range.

Baseline data is reported unless otherwise stated.

Missing data encompassed: 1 for depressive, anxiety, attention-deficit/hyperactivity, defiant and conduct problems; 2 for psychotic experiences; 3 for debt; 6 for somatic problems.

### Debts

To test the predictive value of self-reported psychological symptoms at baseline on debt status at follow-up, hierarchical logistic regression analyses were applied (see Table 2). Higher scores on attention-deficit/hyperactivity problems (odds ratio 1.14, 95% CI 1.03–1.26), oppositional defiant problems (odds ratio 1.18, 95% CI 1.02–1.37) and psychotic experiences (odds ratio 1.12, 95% CI 1.02–1.22) at baseline were predictive of unsecured debts at follow-up, after adjusting only for sex and age. There was no association between depressive, anxiety, somatic or conduct problems and future debts. Adjusting for ethnic background, IQ score and parental psychopathology did not change the findings. Even after controlling for household income, associations remained. Odds ratios for attention-deficit/hyperactivity problems, oppositional defiant problems and psychotic experiences in the fully adjusted model were 1.15 (95% CI 1.04–1.27), 1.17 (95% CI 1.00–1.35) and 1.10 (95% CI 1.01–1.21), respectively. Supplementary Table 2 provides an overview of the regression coefficients of all covariates in the fully adjusted model.

**Table 2:** Outcomes of the hierarchical logistic regression analyses of the association between psychological symptoms at age 15 years and the occurrence of unsecured debts at age 18 years

	Unsecured debts						
	Model 1^a^		Model 2^b^		Fully adjusted model^c^		
	Odds ratio (95% CI)	*P*-value	Odds ratio (95% CI)	*P*-value	Odds ratio (95% CI)	*P*-value	*q*-value
Depressive problems, score	1.06 (0.99–1.14)	0.084	1.05 (0.97–1.13)	0.224	1.05 (0.97–1.13)	0.225	0.106
Anxiety problems, score	1.04 (0.95–1.14)	0.435	1.03 (0.93–1.13)	0.599	1.03 (0.93–1.13)	0.588	0.238
Somatic problems, score^d^	1.38 (0.97–1.95)	0.072	1.37 (0.96–1.95)	0.087	1.39 (0.97–1.99)	0.074	0.052
Attention-deficit/hyperactivity problems, score	1.14 (1.03–1.26)	0.009**	1.14 (1.03–1.27)	0.010*	1.15 (1.04–1.27)	0.008**	0.023*
Oppositional defiant problems, score	1.18 (1.02–1.37)	0.023*	1.16 (1.00–1.35)	0.043*	1.17 (1.00–1.35)	0.043*	0.041*
Conduct problems, score	1.10 (0.99–1.21)	0.067	1.09 (0.98–1.20)	0.105	1.09 (0.98–1.20)	0.099	0.056
Psychotic experiences	1.12 (1.02–1.22)	0.013*	1.10 (1.01–1.21)	0.032*	1.10 (1.01–1.21)	0.033*	0.041*

*q*-values are *P*-values adjusted using Storey’s false discovery rate approach.

a Adjusted for sex and age.

b Additionally adjusted for ethnic background, IQ score and parental psychopathology.

c Additionally adjusted for household income.

d A square root transformation of the somatic problems score was applied to solve the violation of the linearity of the logit assumption.

*P < 0.05, **P < 0.01.

### Experienced financial scarcity

To test the predictive value of self-reported psychological symptoms at baseline on experienced financial scarcity at follow-up, hierarchical linear regression analyses were conducted (see Table 3). More depressive (*B* = 0.05, 95% CI 0.03–0.06), anxiety (*B* = 0.03, 95% CI 0.02–0.05), somatic (*B* = 0.04, 95% CI 0.02–0.07), attention-deficit/hyperactivity (*B* = 0.04, 95% CI 0.02–0.06), oppositional defiant (*B* = 0.06, 95% CI 0.03–0.09) and conduct (*B* = 0.05, 95% CI 0.03–0.07) problems, and psychotic experiences (*B* = 0.05, 95% CI 0.03–0.06) at baseline, were predictors of greater experienced financial scarcity at follow-up, adjusted for sex and age. Associations persisted after adjusting for ethnic background, IQ score and parental psychopathology. Adding household income to the model slightly attenuated the associations, but did not fully account for them. For somatic problems, the association was no longer significant after applying the false discovery rate correction. Supplementary Table 3 presents the regression coefficients of all covariates in the fully adjusted model.

**Table 3:** Outcomes of the hierarchical linear regression analyses of the association between psychological symptoms at age 15 years and the level of experienced financial scarcity at age 18 years

	Experienced financial scarcity, score						*q*-value
	Model 1^a^		Model 2^b^		Fully adjusted model^c^		
	*B* (95% CI)	*P*-value	*B* (95% CI)	*P*-value	*B* (95% CI)	*P*-value	
Depressive problems, score	0.05 (0.03–0.06)	<0.001***	0.04 (0.03–0.05)	<0.001***	0.04 (0.03–0.06)	<0.001***	<0.001***
Anxiety problems, score	0.03 (0.02–0.05)	<0.001***	0.03 (0.01–0.05)	<0.001***	0.03 (0.01–0.05)	<0.001***	0.009**
Somatic problems, score	0.04 (0.02–0.07)	0.001**	0.03 (0.01–0.06)	0.007**	0.04 (0.01–0.06)	0.006**	0.150
Attention-deficit/hyperactivity problems, score	0.04 (0.02–0.06)	<0.001***	0.04 (0.02–0.06)	<0.001***	0.04 (0.02–0.06)	<0.001***	0.004**
Oppositional defiant problems, score	0.06 (0.03–0.09)	<0.001***	0.06 (0.03–0.09)	<0.001***	0.06 (0.03–0.09)	<0.001***	0.005**
Conduct problems, score	0.05 (0.03–0.07)	<0.001***	0.04 (0.02–0.06)	<0.001***	0.04 (0.02–0.06)	<0.001***	0.004**
Psychotic experiences	0.05 (0.03–0.06)	<0.001***	0.04 (0.02–0.06)	<0.001***	0.04 (0.02–0.06)	<0.001***	0.001**

*q*-values are *P*-values adjusted using Storey’s false discovery rate approach. A square root transformation of experienced financial score was applied to solve the violation of the normality of the residuals assumption.

a Adjusted for sex and age.

b Additionally adjusted for ethnic background, IQ score and parental psychopathology.

c Additionally adjusted for household income.

**P < 0.01, ***P < 0.001.

## Discussion

We examined how different self-reported psychological symptoms during mid-adolescence were related to subsequent unsecured debts and experienced financial scarcity at 18 years of age, the starting point of legal financial responsibility. More attention-deficit/hyperactivity problems, oppositional defiant problems and psychotic experiences were associated with increased future occurrence of unsecured debts when entering adulthood. Additionally, more psychotic experiences, depressive, anxiety, somatic, attention-deficit/hyperactivity, oppositional defiant and conduct problems were associated with increased future experienced financial scarcity during emerging adulthood. Importantly, these relations were not explained by lower family household income. Our findings indicate that psychological symptoms during mid-adolescence may precede objective and subjective financial difficulties when becoming legally financially responsible.

The finding that behavioural problems, i.e. attention-deficit/hyperactivity and oppositional defiant problems, were related to future debts aligns with other studies showing a relation between criminal behaviour or attention-deficit/hyperactivity symptoms and later financial problems.^9–12^ One possible explanation is that attention-deficit/hyperactivity disorder and oppositional defiant disorder are linked to increased risk-taking and impulsivity.^36–38^ Indeed, more impulsive buying, substance use and gambling are observed in individuals with attention-deficit/hyperactivity disorder or oppositional defiant problems.^39–44^ Taking on unsecured debts can be seen as another form of impulsive behaviour, and this risk-taking may explain the observed associations. Notably, conduct problems were not related to future debts in our study, which was surprising as oppositional defiant disorder and conduct disorder share similar behaviours.^44^ However, oppositional defiant problems include emotional dysregulation, which is not included in the criteria of conduct disorder,^44^ and together with heightened impulsivity, this may create a higher vulnerability for taking on debts. Our findings also imply a different mechanism for behavioural problems compared with internalising problems, which were not related to future debts. Our findings suggest that specifically attention-deficit/hyperactivity and oppositional deviant problems during mid-adolescence may make an individual more vulnerable for debt through financial risk-taking. Given that other studies showed that financial problems also have a negative effect on psychopathology severity, a vicious cycle may be initiated.

Additionally, the finding that more psychotic experiences at age 15 years were associated with an increased occurrence of later debts is in line with studies among adults, which found a higher prevalence of psychotic disorders in individuals with debts.^5^ Increased impulsivity and risk behaviour may also be relevant in the context of psychotic experiences.^45,46^ Furthermore, psychotic experiences are associated with a range of adverse functional outcomes and overall impairments.^46,47^ This may hinder their ability to manage their finances effectively and may make them vulnerable to future financial instability, such as unsecured debts.

Interestingly, although a link between depression and debts has been demonstrated,^5^ internalising problems (i.e. depressive, anxiety and somatic problems) were not related to later debts in our study. Internalising problems do not seem to serve as a risk factor for financial risk-taking leading to the later accumulation of debts. In line with previous longitudinal studies that solely identified the opposite relation, i.e. financial problems preceding internalising problems,^6^ it could be that objective financial difficulties, such as debts, may induce stress and worries and contribute to the development of internalising symptoms.^5^

In examining subjective financial difficulties, the finding that all types of psychological symptoms, except somatic problems, were associated with subsequently experienced scarcity suggests that psychological symptoms may make adolescents vulnerable to negative appraisals of their finances. Yet, different mechanisms may be involved, depending on the specific symptoms. First, individuals with internalising mental health problems (i.e. depression or anxiety) often exhibit general worrying, rumination and negative thoughts,^22,23^ which may also manifest as financial concerns. Second, adolescents with more attention-deficit/hyperactivity problems, oppositional defiant problems or psychotic experiences had an increased likelihood of debts, indicating that objective financial difficulties are more frequent among these adolescents, which they may also perceive subjectively. For these externalising symptoms, the mechanism might work through increased risk-taking and impulsivity,^36–38,45,46^ which could lead to financial risk-taking as described before, and potentially legitimate concerns about their financial situation. Third, symptoms can result in difficulties performing at school or work;^13,14^ for example, increased absenteeism owing to internalising disorders,^13^ difficulties with learning and attention associated with attention-deficit hyperactivity disorder^48^ or misbehaviour in the context of externalising symptoms.^13^ In any of these cases, these symptoms may put adolescents in a financially vulnerable position, which is reflected in the increased experience of financial scarcity.

### Strengths and limitations

First, we included both an objective and subjective measure of financial difficulties, which revealed different relations. Second, with a high-risk sample drawn from the general population, we had sufficient power for our detailed analyses.^49^ Third, we addressed an important limitation of other studies^6^ by demonstrating that associations persist even when accounting for socioeconomic background, using family income as a proxy. Fourth, by following a sample starting in mid-adolescence and continuing into emerging adulthood, we were able to examine the unique period in which both psychopathology and financial responsibility first emerge.

Despite the strengths of the study, several limitations should be taken into account when interpreting the results. First, we lacked baseline data on adolescents’ financial situation, preventing us from ruling out a reverse longitudinal relation. However, it is unlikely that (significant) debts have started before age 15 years, particularly given the legal framework that protects them from taking on official debts. Yet, in our analyses we assumed a unidirectional pathway, leaving the question of causality unresolved. Also, we analysed the data as independent cross-sectional observations and were unable to model trajectories of change in both psychological symptoms and financial measures, resulting in insufficient understanding of how both measures fluctuate over time and interact with one another. Additionally, considering that debts were still relatively rare at age 18 years, we may have missed more long-term effects. Since debts typically accumulate with age,^50^ a longer follow-up time could reveal different, most likely stronger, effects. Second, despite our high-risk sample, the number of adolescents in debt was too small to conduct analyses that differentiated between the specific types of debts. Different relations may exist for different forms of debt, e.g. study loans or credit card debt. Third, both psychological symptoms and financial measures were self-reported and not objectively verified. Yet, self-report measures provide essential insights into adolescents’ own experiences.^51^ Besides, unsecured debts can remain unregistered, potentially missing the full spectrum of debts. Finally, we were unable to include adolescents that participated without a parent. It is possible that these adolescents may have different characteristics, such as different socioeconomic background or less parental involvement, which may explain the increased occurrence of adolescent debt in the non-included participants, which should be taken into account when generalising the study outcomes.

### Future research

Our research provides leads for future studies to further clarify the relation between psychopathology and financial difficulties. First, studies should focus on both directions in various age groups and model individual trajectories of change in both psychological symptoms and financial difficulties. Together, this will enhance the understanding of how financial difficulties and psychopathology affect each other over the lifespan, taking into account the different dynamics that occur at different life stages. Next, we encourage including subjective financial measures next to objective measures, as different relations and mechanisms may exist. Subjective measures capture how individuals perceive their financial situation, which may differ from their actual situation and can be of particular relevance in light of mental health. Moreover, although we adjusted for family income, we could not examine the role of parental perceived financial stress, which is known to influence children’s (financial) development^52^ and may provide additional context for future findings. Furthermore, it would be interesting to explore how psychopathology is related to different types of unsecured debts. It could be hypothesised that behavioural symptoms may be more strongly related to less commonly accepted debts, such as borrowing from others or unsecured loans from financial institutions, compared with more widely accepted debt sources like student loans. Finally, it could be insightful to investigate the hypothesised different mechanisms that underlie the associations between psychological symptoms and financial problems. As we suggested, risk-taking and impulsivity may serve as an underlying mechanism for the association with externalising symptoms, whereas rumination and cognitive biases may be relevant in the context of internalising symptoms.

### Practical implications

To alleviate financial and mental health challenges during emerging adulthood, we suggest a comprehensive approach that incorporates financial education into existing preventative programmes. Adolescents displaying behavioural problems seem especially prone to adverse financial outcomes, such as debts. Targeted interventions aimed at preventing financial risk-taking behaviours in this group may be beneficial, before these behaviours develop into more severe financial problems. For those with internalising problems but a healthy financial situation, cognitive–behavioural therapy might be effective to address and reduce negative cognitions about their finances. Furthermore, rather than inquiring solely about objective financial problems, it might be more insightful to assess how adolescents with mental health problems perceive their financial situation. Their subjective beliefs may influence their future financial decisions or hinder them from entering mental healthcare because of the perceived costs. Early-onset psychopathology has been associated with a wide range of negative outcomes. Here, we add that it is important to also consider both subjective and objective financial strain in emerging adulthood. An integrated approach should promote long-term financial stability for adolescents facing mental health problems.

## Supporting information

Ravensbergen et al. supplementary material Ravensbergen et al. supplementary material

## Data Availability

The data that support the findings of this study are available on request from the corresponding author, D.C.B. The data are not publicly available due to privacy and ethical restrictions. Other researchers are welcome to collaborate with researchers in the iBerry Study group and to request access to the data. Proposals to collaborate will be assessed by the iBerry Study group with respect to quality, feasibility and potential overlap with planned or published publications.

## Supplementary material

The supplementary material is available online at https://doi.org/10.1192/bjo.2025.10844
