# Supplementary material for: Associations between psychological symptoms in adolescence and debts or experienced financial scarcity in emerging adulthood
Source: BJPsych Open. 2025 Sep 19;11(5):e217. doi: 10.1192/bjo.2025.10844 (PMC12451541; doi:10.1192/bjo.2025.10844)
Supplement: Ravensbergen et al. supplementary material [file S2056472425108442sup001.pdf]

Supplementary Material

Supplementary table S1. Comparison between the complete case sample and cases with missing values in covariates.

	**Complete case (*n*=659)**	**Missingness in covariates (*n*=115)**	
Age, years (*M, SD*)	14.80 (0.79)	15.75 (1.11)	**t=8.80, p<.001**
Sex, female (*n*, %)	355 (53.9%)	57 (49.6%)	$\chi2$=.729, p=.393
Ethnic background, Dutch (*n*, %)	529 (80.3%)	67 (58.8%)	**$\chi2$=25.45, p<.001**
IQ score (*M*, *SD*)	99.55 (13.56)	95.33 (13.63)	**t=−2.68, p=.004**
Household net monthly income (*n*, %)			$\chi2$=3.33, p=.343
<€1600	60 (9.1%)	6 (16.2%)	
€1600-2399	97 (14.7%)	7 (18.9%)	
€2400-4399	349 (53.0%)	15 (40.5%)	
≥€4400	153 (23.2%)	9 (24.3%)	
Parental psychopathology score (*Mdn*, IQR)	5.00 (10.00)	4.00 (6.25)	U=7561.00, p=.730
Depressive problems score (*Mdn*, IQR)	4.00 (5.00)	4.00 (6.00)	U=34407.00, p=.119
Anxiety problems score (*Mdn*, IQR)	3.00 (4.00)	4.00 (4.00)	U=35548.00, p=.298
Somatic problems score (*Mdn*, IQR)	2.00 (4.00)	2.00 (3.00)	U=34904.50, p=.220
Attention deficit/hyperactivity problems score (*Mdn*, IQR)	7.00 (4.00)	6.00 (4.00)	U=40363.50, p=.250
Oppositional defiant problems score (*Mdn*, IQR)	2.00 (3.00)	2.00 (3.00)	U=37348.00, p=.823
Conduct problems score (*Mdn*, IQR)	3.00 (3.00)	3.00 (3.00)	U=36249.00, p=.469
Psychotic experiences (*Mdn*, IQR)	3.00 (4.00)	3.00 (3.00)	U=34815.50, p=.227
Debts present at follow-up, yes (*n*, %)	51 (7.8%)	19 (16.7%)	**$\chi2$=9.29, p=.002**
Experienced financial scarcity at follow-up, score (*Mdn*, IQR)	2.00 (4.00)	2.00 (4.00)	U=37154.00, p=.850

Note. Missing data encompassed: 1 in ethnic background, experienced financial scarcity, depressive, anxiety, attention deficit/hyperactivity, oppositional defiant, and conduct problems; 3 in psychotic experiences; 4 in debts; 6 in somatic problems; 78 in household income; and 93 in parental psychopathology.
Significant differences are printed in bold.
Abbreviations: IQR=Interquartile range; *M*=Mean; *Mdn*=Median; *SD*=Standard deviation

Supplementary Table S2. Estimates of the fully adjusted logistic regression models of the association between psychological symptoms at age fifteen and the occurrence of unsecured debts at age eighteen.

	OR (95% CI)	*p*-value
**Depressive problems**	1.05 (0.97;1.13)	.225
Age	0.86 (0.59;1.26)	.448
Sex, female	1.11 (0.61;2.04)	.733
Ethnic background, Dutch	**2.46 (1.30;4.65)**	**.006**
IQ score	1.01 (0.99;1.03)	.408
Parental psychopathology	1.02 (0.99;1.04)	.228
Household income, €1600–€2399	0.46 (0.14;1.53)	.203
Household income, €2400–€4399	0.80 (0.32;2.00)	.628
Household income, ≥€4400	0.80 (0.28;2.29)	.680
**Anxiety problems**	1.03 (0.93;1.13)	.588
Age	0.87 (0.60;1.28)	.488
Sex, female	1.16 (0.63;2.12)	.629
Ethnic background, Dutch	**2.52 (1.33;4.76)**	**.004**
IQ score	1.01 (0.99;1.03)	.449
Parental psychopathology	1.02 (0.99;1.05)	.160
Household income, €1600–€2399	0.45 (0.13;1.50)	.192
Household income, €2400–€4399	0.77 (0.31;1.93)	.581
Household income, ≥€4400	0.77 (0.27;2.19)	.627
**Somatic problems***	1.39 (0.97;1.99)	.074
Age	0.80 (0.54;1.18)	.264
Sex, female	1.07 (0.57;1.99)	.841
Ethnic background, Dutch	**2.47 (1.29;4.73)**	**.006**
IQ score	1.01 (0.99;1.03)	.416
Parental psychopathology	1.02 (0.99;1.05)	.172
Household income, €1600–€2399	0.52 (0.15;1.80)	.302
Household income, €2400–€4399	0.97 (0.37;2.54)	.948
Household income, ≥€4400	0.99 (0.33;2.95)	.988
**Attention deficit/hyperactivity problems**	**1.15 (1.04;1.27)**	**.008**
Age	0.94 (0.64;1.39)	.764
Sex, female	1.24 (0.68;2.25)	.479
Ethnic background, Dutch	**2.56 (1.36;4.83)**	**.004**
IQ score	1.01 (0.99;1.03)	.357

Parental psychopathology	1.02 (0.99;1.04)	.188
Household income, €1600–€2399	0.41 (0.12;1.37)	.146
Household income, €2400–€4399	0.72 (0.29;1.78)	.472
Household income, ≥€4400	0.73 (0.26;2.05)	.546
**Oppositional defiant problems**	**1.17 (1.00;1.35)**	**.043**
Age	0.86 (0.59;1.27)	.452
Sex, female	1.19 (0.66;2.16)	.561
Ethnic background, Dutch	**2.47 (1.30;4.68)**	**.006**
IQ score	1.01 (0.99;1.03)	.448
Parental psychopathology	1.02 (0.99;1.05)	.178
Household income, €1600–€2399	0.46 (0.14;1.54)	.207
Household income, €2400–€4399	0.81 (0.32;2.04)	.653
Household income, ≥€4400	0.82 (0.28;2.35)	.707
**Conduct problems**	1.09 (0.98;1.20)	.099
Age	0.86 (0.58;1.25)	.425
Sex, female	1.30 (0.71;2.36)	.397
Ethnic background, Dutch	**2.50 (1.32;4.73)**	**.005**
IQ score	1.01 (0.99;1.03)	.417
Parental psychopathology	1.02 (0.99;1.05)	.177
Household income, €1600–€2399	0.45 (0.13;1.50)	.194
Household income, €2400–€4399	0.79 (0.32;1.99)	.620
Household income, ≥€4400	0.81 (0.28;2.31)	.692
**Psychotic experiences**	**1.10 (1.01;1.21)**	**.033**
Age	0.87 (0.60;1.28)	.485
Sex, female	1.16 (0.64;2.11)	.616
Ethnic background, Dutch	**2.47 (1.30;4.68)**	**.006**
IQ score	1.01 (0.99;1.03)	.399
Parental psychopathology	1.02 (0.99;1.04)	.241
Household income, €1600–€2399	0.45 (0.14;1.52)	.200
Household income, €2400–€4399	0.77 (0.31;1.91)	.567
Household income, ≥€4400	0.81 (0.29;2.30)	.692

Note. Significant results are printed in bold.

* A square root transformation of the somatic problems score was used.

Abbreviations: CI = Confidence interval, OR = odds ratio.

Supplementary Table S3. Estimates of the fully adjusted linear regression models of the association between psychopathology problems at age fifteen and the level of experienced financial scarcity at age eighteen.

	OR (95% CI)	*p*-value
**Depressive problems**	**0.04 (0.03;0.06)**	**<.001**
Age	**−0.07 (−0.14;0.00)**	**.042**
Sex, female	0.01 (−0.10;0.11)	.914
Ethnic background, Dutch	**−0.07 (−0.14;0.00)**	**.042**
IQ score	**−0.01 (−0.01;0.00)**	**.001**
Parental psychopathology	0.01 (0.00;0.01)	.073
Household income, €1600–€2399	−0.03 (−0.25;0.20)	.816
Household income, €2400–€4399	0.01 (−0.19;0.20)	.948
Household income, ≥€4400	0.05 (−0.16;0.26)	.656
**Anxiety problems**	**0.03 (0.01;0.05)**	**<.001**
Age	−0.06 (−0.13;0.01)	.082
Sex, female	0.03 (−0.08;0.14)	.595
Ethnic background, Dutch	**0.24 (0.10;0.37)**	**.001**
IQ score	**−0.01 (−0.01;0.00)**	**<.001**
Parental psychopathology	**0.01 (0.00;0.01)**	**.023**
Household income, €1600–€2399	−0.04 (−0.27;0.19)	.731
Household income, €2400–€4399	−0.01 (−0.21;0.18)	.896
Household income, ≥€4400	0.01 (−0.20;0.23)	.901
**Somatic problems**	0.04 (0.01;0.06)	.006*
Age	−0.06 (−0.13;0.01)	.076
Sex, female	0.03 (−0.09;0.14)	.640
Ethnic background, Dutch	**0.23 (0.09;0.37)**	**.001**
IQ score	**−0.01 (−0.01;0.00)**	**.002**
Parental psychopathology	**0.01 (0.00;0.01)**	**.009**
Household income, €1600–€2399	−0.03 (−0.26;0.19)	.771
Household income, €2400–€4399	0.01 (−0.19;0.21)	.945
Household income, ≥€4400	0.03 (−0.19;0.24)	.819
**Attention deficit/hyperactivity problems**	**0.04 (0.02;0.06)**	**<.001**
Age	−0.04 (−0.11;0.03)	.308
Sex, female	0.07 (−0.03;0.18)	.175
Ethnic background, Dutch	**0.23 (0.10;0.37)**	**.001**
IQ score	**−0.01 (−0.01;0.00)**	**.001**

Parental psychopathology	**0.01 (0.00;0.01)**	**.013**
Household income, €1600–€2399	−0.05 (−0.28;0.17)	.657
Household income, €2400–€4399	−0.03 (−0.23;0.17)	.759
Household income, ≥€4400	0.00 (−0.21;0.22)	.977
**Oppositional defiant problems**	**0.06 (0.03;0.09)**	<.001
Age	−0.06 (−0.13;0.01)	.100
Sex, female	0.06 (−0.04;0.17)	.253
Ethnic background, Dutch	**0.22 (0.08;0.36)**	**.001**
IQ score	**−0.01 (−0.01;0.00)**	**.001**
Parental psychopathology	**0.01 (0.00;0.01)**	**.009**
Household income, €1600–€2399	−0.04 (−0.27;0.19)	.723
Household income, €2400–€4399	−0.02 (−0.22;0.18)	.856
Household income, ≥€4400	0.01 (−0.20;0.23)	.896
**Conduct problems**	**0.04 (0.02;0.06)**	<.001
Age	−0.06 (−0.13;0.01)	.083
Sex, female	0.10 (−0.01;0.21)	.063
Ethnic background, Dutch	**0.23 (0.09;0.36)**	**.001**
IQ score	**−0.01 (−0.01;0.00)**	**.001**
Parental psychopathology	**0.01 (0.00;0.01)**	**.012**
Household income, €1600–€2399	−0.04 (−0.27;0.19)	.733
Household income, €2400–€4399	−0.02 (−0.22;0.18)	.836
Household income, ≥€4400	0.02 (−0.19;0.24)	.848
**Psychotic experiences**	**0.04 (0.02;0.06)**	<.001
Age	−0.05 (−0.12;0.02)	.140
Sex, female	0.06 (−0.04;0.17)	.249
Ethnic background, Dutch	**0.22 (0.09;0.36)**	**.001**
IQ score	**−0.01 (−0.01;0.00)**	**.001**
Parental psychopathology	**0.01 (0.00;0.01)**	**.018**
Household income, €1600–€2399	−0.02 (−0.25;0.20)	.838
Household income, €2400–€4399	−0.02 (−0.21;0.18)	.870
Household income, ≥€4400	0.03 (−0.18;0.25)	.750

Note. Significant results are printed in bold.
The square root transformed score of experienced financial scarcity was used.
* No longer significant after false discovery rate correction.
Abbreviations: CI = Confidence interval, OR = odds ratio.
